# Collaborative governance in the Quebec Cancer Network: a realist evaluation of emerging mechanisms of institutionalization, multi-level governance, and value creation using a longitudinal multiple case study design

**DOI:** 10.1186/s12913-019-4586-z

**Published:** 2019-10-25

**Authors:** Dominique Tremblay, Nassera Touati, Thomas Poder, Helen-Maria Vasiliadis, Karine Bilodeau, Djamal Berbiche, Jean-Louis Denis, Marie-Pascale Pomey, Johanne Hébert, Geneviève Roch, Catherine Prady, Lise Lévesque

**Affiliations:** 1 Faculté de médecine et des sciences de la santé, Campus de Longueuil - Université de Sherbrooke, 150 Place Charles-Le Moyne, Longueuil, Québec, J4K 0A8 Canada; 2 Centre de recherche Charles-Le Moyne - Saguenay–Lac-Saint-Jean sur les innovations en santé (CR-CSIS), Centre intégré de santé et de services sociaux de la Montérégie-Centre, 150 Place Charles-Le Moyne, Longueuil, Québec, J4K 0A8 Canada; 3 École Nationale d’Administration Publique, 4750 Henri-Julien Avenue, Montréal, Québec, H2T 3E5 Canada; 4 Département de gestion, d’évaluation et de politique de santé, École de santé publique, Université de Montréal, 7101, avenue du Parc, 3e étage, Montréal, Québec, H3N 1X9 Canada; 5 Centre de recherche de l’Institut Universitaire en Santé Mentale de Montréal, 7331, rue Hochelaga, Montréal, Québec, H1N 3V2 Canada; 6 École de gestion, Université de Sherbrooke, 2500, boulevard de l’Université, Sherbrooke, Québec, J1K 2R1 Canada; 7 Centre de recherche du Centre Hospitalier de l’Université de Sherbrooke (CR-CHUS), 3001, 12e Avenue Nord, Sherbrooke, Québec, J1H 5N4 Canada; 8 Faculté des sciences infirmières, Université de Montréal, 2375 chemin Côte-Ste-Catherine, Montréal, Québec, H3T 1A8 Canada; 9 Centre de recherche du Centre hospitalier de l’Université de Montréal (CR-CHUM), 850, rue Saint-Denis, Montréal, Québec, H2X 0A9 Canada; 10 École de santé publique, Université de Montréal, 7101, avenue du Parc, Montréal, Québec, H3N 1X9 Canada; 11 Centre de recherche en droit public, Université de Montréal, 3101, chemin de la Tour, Montréal, Québec, H3T 1J7 Canada; 12 Institut de recherche en santé publique de l’Université de Montréal (IRSPUM), Université de Montréal, 7101, avenue du Parc, Montréal, Montréal, Québec, H3N 1X9 Canada; 13 Département des sciences infirmières, Campus de Lévis - Université du Québec à Rimouski (UQAR, 1595, boulevard Alphonse-Desjardins, Lévis, Québec, G6V 0A6 Canada; 14 Hôtel-Dieu de Lévis, Centre de recherche du CISSS de Chaudière-Appalaches, 143, rue Wolfe, Lévis, Québec, G6V 3Z1 Canada; 15 Centre de recherche du Centre hospitalier universitaire de Québec (CRCHUQ), 11 Côte du Palais, Québec, Québec, G1R 2J6 Canada; 16 Équipe de recherche Michel-Sarrazin en oncologie psychosociale et soins palliatifs (ERMOS), Maison Michel-Sarrazin, 9, rue McMahon, Québec, Québec, G1R 3S3 Canada; 17 Faculté des sciences infirmières, Université Laval, 1050, avenue de la Médecine, Pavillon Ferdinand-Vandry, Québec, Québec, G1V 0A6 Canada; 18 Axe Santé des populations et pratiques optimales en santé, Centre de recherche du CHU de Québec-Université Laval, 10, rue de l’Espinay, Québec, Québec, G1L 3L5 Canada; 19 Centre de recherche sur les soins et les services de première ligne de l’Université Laval (CERSSPL‐UL), 2525, chemin de la Canardière, Québec, Québec G1J 0A4 Canada; 20 Centre intégré de santé et de services sociaux de la Montérégie-Centre, 3120 boulevard Taschereau, Greenfield Park, Québec, J4V 2H1 Canada; 21 Centre intégré de cancérologie de la Montérégie, 3120 Boulevard Taschereau, Greenfield Park, Québec, J4V 2G9 Canada

**Keywords:** Network governance, Patient care experience, Resource utilization, Cost analysis, Longitudinal case study, Realist evaluation, Organizational research

## Abstract

**Background:**

People living with and beyond cancer (PLC) receive various forms of specialty care at different locations and many interventions concurrently or over time. They are affected by the operation of professional and organizational silos. This results in undue delays in access, unmet needs, sub-optimal care experiences and clinical outcomes, and human and financial costs for PLCs and healthcare systems.

National cancer control programs advocate organizing in a network to coordinate actions, solve fragmentation problems, and thus improve clinical outcomes and care experiences for every dollar invested. The variable outcomes of such networks and factors explaining them have been documented. Governance is the “missing link” for understanding outcomes. Governance refers to the coordination of collective action by a body in a position of authority in pursuit of a common goal. The Quebec Cancer Network (QCN) offers the opportunity to study in a natural environment how, why, by whom, for whom, and under what conditions collaborative governance contributes to practices that produce value-added outcomes for PLCs, healthcare providers, and the healthcare system.

**Methods/design:**

The study design consists of a longitudinal case study, with multiple nested cases (4 local networks nested in the QCN), mobilizing qualitative and quantitative data and mixed data from various sources and collected using different methods, using the realist evaluation approach. Qualitative data will be used for a thematic analysis of collaborative governance. Quantitative data from validated questionnaires will be analyzed to measure relational coordination and teamwork, care experience, clinical outcomes, and health-related health-related quality of life, as well as a cost analysis of service utilization. Associations between context, governance mechanisms, and outcomes will be sought. Robust data will be produced to support decision-makers to guide network governance towards optimized clinical outcomes and the reduction of the economic toxicity of cancer for PLCs and health systems.

## Background

People living with and beyond cancer (PLC) need timely access to proven, coordinated, continuous care focused on their values and preferences [1–3]. They must receive care and services, concurrently or over time, from multiple professionals and practitioners working in various locations (ambulatory oncology clinics, hospitalization units, doctors’ offices, CLSCs, homes, palliative care residences) [4, 5]. In order to meet these needs, it is imperative to avoid operating in silos, which results in unwanted outcomes, as has been widely documented [2, 6]. International experience [7, 8], the experience in Quebec (Canada) [9], as well as our previous work [10–12] show that, although structural levers are necessary to integrate care and services, they do not suffice to optimize the supply of services to PLCs. This is why providing safe and high-quality care and services requires a shift from a logic based on autonomy and independence to a logic of interdependence where teamwork allows for the exchange of knowledge and expertise [13] and for shared leadership that goes beyond the invisible walls between professions and organizations, and that includes PLCs [14, 15]. Organizing in the form of an integrated network centred on PLCs is therefore a logical choice.

### A network to integrate care

A network refers to a form of organization of collective action where groups of actors with their own and often competing goals, values, needs, and forms of representation must rally around a common goal [4]. In general, a network is defined as “multiple organizations which are tied by some form of structural interdependence in which one unit is not the subordinate of others [...] that coordinate their joint activities through different types of peer-to-peer relations” ([4] p. 529).

Integrated network operation has been a dominant theme in national cancer control programs in several countries for more than two decades [2, 16–22]. Cancer networks are intended to be a solution to the classic fragmented service offering model, which is not optimal to meet the needs and expectations of PLCs. In recognizing the interdependence inherent in providing cancer care [13], networks are integrative tools to rebuild practices around new relationships essential to the quality and safety of care, thereby reducing the burden of disease.

Studies on health networks have focused mainly on mandated networks, i.e. networks with a political mandate. The multiple factors influencing their implementation are well documented [4, 23–25]: coordination mechanisms and tools, size of the network, internal stability), operational characteristics (manager competence, knowledge sharing, development of innovation capacities, mobilization of professionals, member participation and commitment) and contextual characteristics (stability of the health system, access to human, financial, informational resources, cohesion, community support). Certain studies have concluded that professional commitment, legitimacy leadership, and trust are determinants of network performance and sustainability [24, 26, 27].

Some benefits of integrated network operation have been reported [28]. The most robust evidence comes from networks for elderly persons. Positive outcomes have been demonstrated in terms of improved functional and cognitive status [29–33], access to social support, and satisfaction [30]. However, the benefits for healthcare systems are mixed. Some studies have found a decrease in hospitalizations and readmissions [34, 35], length of hospital stay [36], and use of emergency services [34]. While some studies have reported a decrease in hospital costs, others have concluded that networks have not reduced costs [37].

On the other hand, unanticipated or unwanted results of mandated networks have also been reported. There are always gaps between a network “prescribed” at the political level and one that is taking shape on the ground. Networking is a major change that is not easily imposed, with professionals having a central role in transforming the provision of care [38] and the power to resist the prescription openly or passively [39]. As a result, anticipated benefits of networks are not materializing as expected. These differences can be explained by several individual, organizational, and contextual factors whose influence varies according to the type of network, its stage of development, or its degree of integration [4, 7, 40–44].

For example, Addicott et al. have shown that formalization of the roles of actors (individuals or organizations) in networks could weaken existing informal links before new linking mechanisms can be put in place. This results in dilution of clinical expertise due to dismantling of informal mechanisms for exchange of information and knowledge [45]. This same study also showed that the negative perception of the presence of an external control body in the network undermined managers’ efforts to support clinicians’ ownership of network practices. One of the few studies in the field of cancer care, conducted in the United Kingdom [45], showed how a governance body at the political level focused on organizational restructuring and performance rather than knowledge sharing can have an undesirable impact on the implementation of a network. Others have reported an increase in the use of emergency services due to increased patient vigilance in the event of a deterioration in their state of health and the lack of alternatives [36]. Yet others have found that the resources and efforts devoted to setting up clinical networks generate costs that do not necessarily translate into concrete benefits for service users [40].

### From prescribed network to actual network

The transition from the “prescribed” network (following a national cancer plan) to an “actual” network (in the field) is particularly challenging. Clinicians’ professional practices are based on their autonomy, expertise, identity, and relationships with different stakeholders [46], while clinical decision-making is rooted in the uniqueness of a PLC’s trajectory, which poses challenges for collective action [47]. For managers, being in a network requires significant relational work between professionals and organizations [48] to ensure coordination of care provision in and between different teams, in a system where reforms are affecting all dimensions of cancer care provision [1]. Updating to new approaches that have been conceived at the political level but need to be implemented at the clinical level represents a test of managerial leadership. The challenge is all the greater since the benefits of integrated network operation manifest as small changes in practices and small gains that can take up to 20 years to become visible [49]. However, if professionals do not see the benefits of their transformation efforts, they are likely to be reluctant to change their practice in favour of networking. For policy makers, who are more familiar with hierarchical or “top-down” functioning, networking requires them to learn a new way of making decisions that is shared with non-governmental actors (clinicians, managers, PLCs). As for PLCs, our work shows that they associate their unmet needs in part with the fragmented organization of care and services, and the notion of a network can be abstract for them [50, 51].

### From network governance to collaborative governance

Converging evidence points to the importance of understanding governance mechanisms as factors influencing the evolution of networks and their effectiveness [41]. Governance is increasingly recognized as the “missing link” for understanding networks and their outcomes [52], particularly in the health sector [11, 41, 53–55]. Governance is a relatively new, multi-dimensional concept, lacking a consensus definition [56]. Overall, governance refers to the coordination of collective action by an entity in a position of authority [57]. Effective governance requires the efforts of multiple actors to coordinate with each other by mobilizing different operational rules and strategies to achieve the objectives of a network [58, 59]. A thorough understanding of the mechanisms by which networks achieve their outcomes is necessary to improve cancer management and optimize the use of resources invested in networking. In this case, a better consideration of governance dynamics to support the transformation of practices in favour of a high-quality, affordable, and PLC-focused service offer is promising [41, 53, 56, 58, 60, 61].

Network governance, according to Provan’s foundational work, can be conceptualized in two forms: shared governance and centralized governance [23]. Shared governance is the most flexible and least hierarchical form, where the different partners share decision-making power and interact informally. Centralized governance is provided by a lead organization that assumes the lead role due to its central position in providing services to clients and its authority over the distribution of resources [23]. In a literature review, Turrini et al. concluded that the presence of a coordinating body exercising external control was positively associated with the ability to achieve the objectives pursued by a network [4]. A study by Denis et al. showed the positive impact of consolidating a strong central authority within the network when establishing health and social services centres in Quebec [62].

Without denying the contribution of a strong central body, whether internal or external to the network, the plurality of decision-making forums in knowledge-based professional organizations calls for collaborative governance (CG) of networks to overcome barriers to operating in professional and organizational “silos”, which are detrimental since they increase the risk of adverse events related to coordination dysfunctions in the healthcare system [63]. Among the many definitions of CG, the one that we believe is most useful in the context of cancer networks is as follows: “the processes and structures of public policy decision making and management that engage people constructively across the boundaries of public agencies, levels of government, and/or the public, private and civic spheres in order to carry out a public purpose that could not otherwise be accomplished” [64].

Although CG offers a promising solution, very little work has been done to provide a thorough understanding of the dynamics specific to CG in cancer control. Considering the unprecedented health services reforms conducted not only in Quebec, but in most industrialized countries [1, 63, 65, 66], gaining this understanding is of the utmost importance. Although there are different interpretive frameworks for governance, most are based on theoretical inferences [55, 64] that need to be empirically tested. Aside from the work we are doing on the functions of governance [67], we have not identified any studies that could guide the collaboration of multi-level governance actors in addressing what some jurisdictions call the “cancer crisis” [65]. The present study will thus contribute to addressing the need to identify which governance models are most useful and in which context, and to provide examples of best practices to support decision-making confronted with service fragmentation issues [54].

### Empirical context

Integrated network operations is one of the key elements of the national cancer control program (Programme Québécois de Lutte Contre le Cancer) [2], which lays the foundations for the governance of the Quebec Cancer Network (QCN) to deal with the complexity of care provision [68] (p. 36) while optimizing the use of resources [68] (p. 37). The QCN is a “network of networks where the organization of direct and associative links are both formal and informal. The provincial cancer plan reflects a desire to promote and support local initiatives for networking. To do this, it is necessary to rethink network governance by taking into account clinical and human issues to encourage and equip the capacity to act together (collaboration, coordination). Indeed, the implementation of the QCN poses challenges related to professional issues [69, 70], clinical and bureaucratic routines that create an “iron cage” around practices [71]. Thus, the QCN, a provincial network of local and regional networks, is a typical example where multiple actors (PLCs, clinicians, managers, politicians) with different motivations and acting at different levels of the health system (clinical, organizational, political) must coordinate with each other [72]. As with other clinical client networks, some benefits are observed, but the results are not yet as good as expected [28]. Considering the resources and efforts devoted to governance in the QCN, it is imperative to better understand its mechanisms and implications at the clinical, organizational, political, and economic levels. In addition, the current state of knowledge calls for a better understanding of the outcomes these factors produce for patients [10, 67].

The present study builds upon this research [10, 67] and aims to provide an in-depth analysis of how, why, by whom, for whom, and under what conditions CG in a cancer network contributes to outcomes that have added value for PLCs, providers, and the healthcare system. GC is approached as an innovative intervention with clinical, organizational, and political components that mobilize multiple QCN actors acting at different levels of PLC care delivery. Our working hypotheses are that GC in a cancer network is associated with 1) a more positive perception of the PLC care experience; 2) a more positive perception of health-related quality of life; and 3) clinical benefits. Given the state of knowledge about costs, we adopt a more exploratory approach in assessing whether the degree of GC is associated with cost changes.

Our approach uses as an empirical example the provision of care and services to persons living with and beyond cancer (PLC) in the Quebec Cancer Network (QCN). More specifically, the objectives of the study are to: 1) Identify the critical contextual factors that promote or hinder GC at the clinical, management, and policy levels; 2) Explain how and why CG is activated to materially evolve a cancer network; and 3) Compare the outcomes of network CG on: 3a) care experience, 3b) clinical outcomes, 3c) health-related quality of life, and 3d) costs associated with the use of care and services.

The objectives call for a formative evaluation of CG in the context of hybrid networks such as the QCN – a contemporary phenomenon taking shape in a natural environment – to assess its benefits, which is consistent with the realist evaluation approach [73]. They also involve deepening the analysis of governance mechanisms by focusing more on the interaction between levels of governance [61] and on the interaction between mechanisms of engagement, motivation of actors, and development of capacities for action. Realist evaluation is increasingly being used to understand complex interventions, of which GC is a typical example. In this case, the QCN [68, 74] offers a unique opportunity to submit theoretical GC frameworks to empirical testing in order to draw useful observations for practice.

## Methods/design

### Study design

**Fig. 1:**
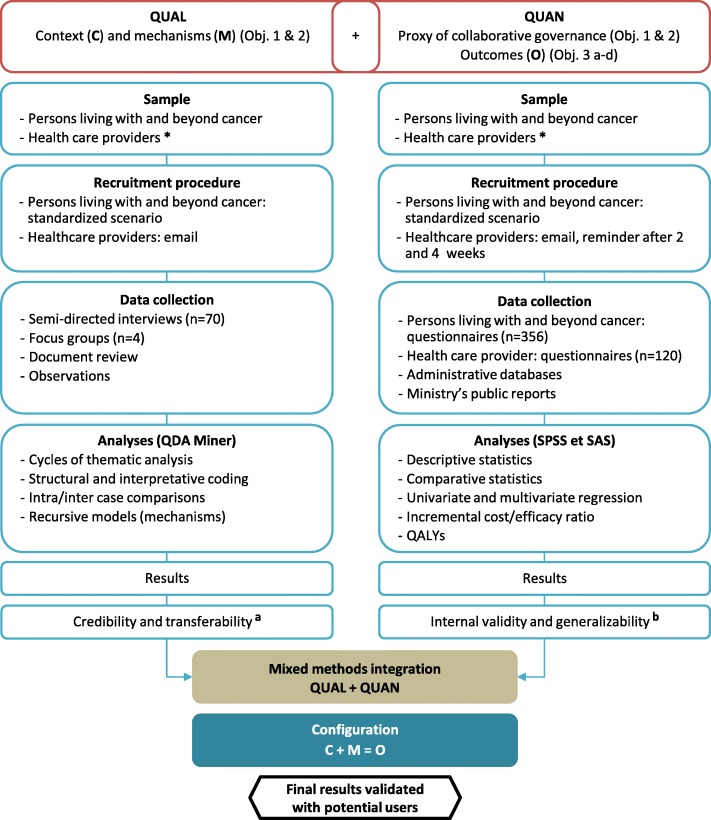
Study design: Multiple nested case study using concurrent mixed methods approaches. *: Health care providers: clinicians, health managers, policy makers involved in provision and delivery of care and services to persons living with cancer; C: Context; M: Mechanisms; O: Outcomes; QUAL: qualitative data; QUAN: quantitative data. *References*: a: Miles MB, Huberman M, Saldana J. Qualitative data analysis. A methods sourcebook. 3rd ed. Thousand Oaks, CA: SAGE Publications; 2014. b: Creswell J. Research design: qualitative, quantitative, and mixed methods approaches. 4 ed. Thousand Oaks, CA: SAGE Publications; 2013

The study design consists of a longitudinal, multiple nested case study [75] using both qualitative and quantitative approaches. The case study, as a methodological approach, is particularly appropriate when the analysis focuses on the dynamics of interaction between actors involved in an intervention in a given context [76]. This design is consistent with realist evaluation [77], which structures our in-depth investigation of the links between CG mechanisms and outcomes (see the study design in Fig. 1). According to realist evaluation, the association between GC contextual factors (C) and mechanisms (M) creates the conditions for the production of outcomes (E). These associations formulated as C + M = E make explanatory models for studying the production of outcomes of complex interventions intelligible. Quantitative data collection will be conducted using concurrent mixed methods approaches to complement qualitative data (Objectives 1,2) and measure the outcomes associated with GC (Objective 3) [78].

### Settings

The cases under study are four Centres intégrés de santé et services sociaux (integrated health and social services centres – CISSS, or CIUSSS for those with an academic mandate) that provide care and services to PLCs. These four local cancer networks, embedded within the QCN, are instrumental in understanding CG in the QCN and increase the explanatory power by specifying the meaning that actors give to their GC practices in their context [76]. They have been chosen in a reasoned way for their informative potential and diversity [79] that will allow us to compare the differences and similarities explaining the variations in CG mechanisms and outcomes in different contexts. Their organizational characteristics are described in Table 1.

**Table 1:** Characteristics of the cancer networks participating in the study

Cancer network (microcase)	1	2	3	4
Geographic location	Rural/Semi-urban area	Metropolitan area	Metropolitan area	Rural/Semi-urban/Urban area
Surface (km^2^)	> 10,000	< 100	< 100	> 1000
Population (*N*)	> 400,000	< 400,000	> 400,000	< 400,000
Cancer services offered				
Radiotherapy	As per inter-regional service agreement	In the region	In the region	At the Integrated Cancer Centre
Integrated Cancer Centre	Launched	No	No	Yes
Networked healthcare settings				
Hospitals (N)	5	2	3	2
Community health centres^a^ (N)	26	8	6	7
Family medicine groups^b^ (N)	39	7	12	10
Network development stage	Emergent	Emergent	Intermediary	Mature

^a^Centre local de services communautaires (CLSC); ^b^Include Groupe de médecine de famille (GMF); Unités de médecine familiale (UMF), et Groupe de médecine de famille universitaire (GMF-U)

### Participants

The inclusion criteria under study for caregivers are: being a health professional, manager, policy maker, or a PLC representative involved in governance and, directly or indirectly, in the provision of care to PLCs. All participants will sign a consent form approved by a recognized research ethics board. Throughout the study, ethical considerations will be addressed through a relational and collaborative approach, particularly with study participants, including the PLCs [80, 81].

The inclusion criteria for PLCs are: having a confirmed diagnosis of a type of cancer (lung, breast, colorectal, prostate) selected based on the number of new cases and the toxicity of the treatment that may have an impact on health-related quality of life [82], reading and understanding French, having a care experience of at least two cancer treatments or early palliative (non-end of life) treatments, and having the physical and cognitive capacity to answer a questionnaire of about 20 min. This capacity will be determined in collaboration with clinicians who know the potential participants.

### Qualitative data collection

The collection of qualitative data from various sources and methods [75] will focus on contextual factors and mechanisms surrounding CG in the QCN as perceived by stakeholders (Objectives 1, 2). The triangulation of sources and methods should compensate for their respective inherent limitations [83] and contribute to the study’s internal validity [84].

#### Field documents

The document review will be conducted to form an initial idea of what is happening in the cancer networks with respect to CG. Documents collected will include meeting minutes, action plans, regional and government reports, and scientific literature.

#### Focus groups

Heterogeneous focus groups of six to eight participants per case [85], selected on the basis of intentional sampling based on their role in the QCN and their knowledge of governance issues, will be formed. Care providers in the roles of medical and clinical-administrative co-manager, cancer services representative, performance evaluation manager, or PLC representatives will be invited to participate. The facilitation (*n* = 4) will be led by an experienced person with excellent knowledge of cancer care and services.

#### Interviews

After a first cycle of document analysis, semi-structured individual interviews will be conducted with key informants and representatives of government departments involved in cancer research (public health, primary care, medical care, and nursing). Based on our previous studies and the literature [86, 87], approximately 15 participants per case and about 10 at the provincial level (total *n* = 70) will be required. This number will be adjusted according to the nature and redundancy of the data [87].

#### Data collection grids

**Fig. 2:**
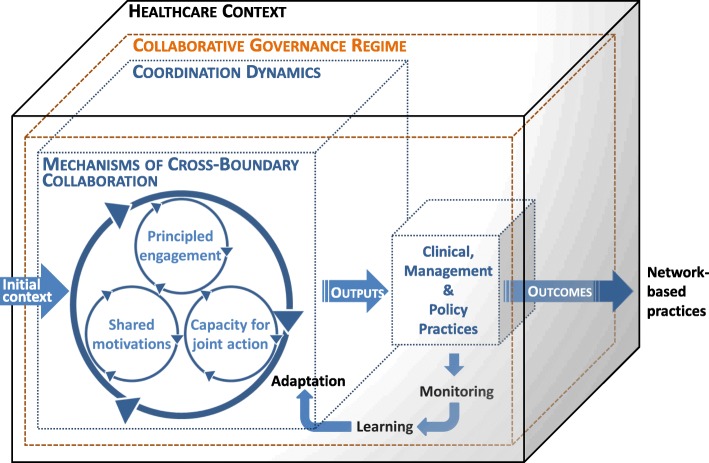
Cancer network collective governance framework. The framework has been adapted from Emerson [64, 89] and integrated with Pawson and Tilley’s concepts [73]

Emerson’s framework [64], itself based on the founding work of Ansell and Gash [88], has been adapted to guide the collection of qualitative data on CG mechanisms and their outcomes (Fig. 2 and Additional file 1). Emerson et al. explicitly defined the components of their model [64, 89]. Data collection grids, developed from the concepts of this analytical framework, will be used and their content adapted according to the nature of the information sought and the type of actors. The data sought will focus on the characteristics of the context (history, opportunities, constraints), the characteristics of the actors (values, leadership, management strategies, resources), the dynamics of collaboration (instruments, strategies, evolution over time), the perceived influence of these dynamics on the care experience, the health-related quality of life of PLCs, and the costs related to the use of services. Participants will also be asked to describe facts or incidents, expected or not, that have more critically influenced CG and the resulting collaborative dynamics.

### Qualitative analysis

The audio data recorded during the interviews and focus groups will be transcribed and stored in a formal database managed using QDA Miner Software version 4.1.21 [90]. Analysis of qualitative data will begin as soon as the first data become available and will follow cyclical thematic analysis techniques based on our analytical framework: structural coding followed by interpretive analysis [91]. The intra-case analysis will be followed by an inter-case analysis to contrast the differences and similarities in the recursive mechanisms of CG from a vertical perspective (between levels of governance) and horizontal perspective (between and within teams). Associations between critical contextual factors and collaborative governance mechanisms will be constructed according to the principles of realist evaluation [73] and our research objectives. The validity of the QUAL results will be ensured by the use of an analytical framework with theoretical foundations (Additional file 1), multiple triangulation [92] of data sources, methods and researchers with varied expertise, and the involvement of collaborators and PLCs to ensure the plausibility of findings. QUAL+QUAN data will be integrated using a convergent approach to highlight differences and allow some combination of QUAL+QUAN results [78]. Transferability will be maximized by developing a rich description of contexts and mechanisms [91].

### Quantitative data collection

The quantitative data collected will allow the quantification of GC outcomes on care providers and PLCs as well as the financial burden of cancer in terms of expenditures incurred by PLCs and the cost to the healthcare system of the services used. The data collection will be based on a concurrent mixed methods approach to complement the qualitative data (Objectives 1, 2) and measure the outcomes associated with CG (Objective 3) [78].

#### Health care provider questionnaires

The description of the questionnaires that will be used for the collection is presented in Additional file 2. Relational coordination [93], Practice Environment Checklist (PEC) [94] and teamwork [95] are operational proxies at the clinical and organizational level for the desired CG outcomes for care providers. The sample size of the caregivers should be 30 for each case (total *n* = 4*30 = 120) considering a minimum response rate of 35%. The inclusion criteria are: being a professional or manager involved in governance and directly or indirectly involved in providing care to PLCs.

The questionnaire will be self-administered electronically on SurveyMonkey to individuals directly or indirectly involved in the provision of care to PLCs in each case as well as to individuals involved at the political level (MSSS and provincial coordinating committee).

#### Questionnaires for persons living with and beyond cancer

Outcome measurement for PLCs includes care experience, health-related quality of life, clinical information and expenses incurred as perceived by PLCs (patient-reported experience measure) [96] (Additional file 2). The care experience will be operationalized by the Health System Responsiveness questionnaire [97], health-related quality of life by the EuroQoL EQ-5D-5 L questionnaire and analogous visual scale [98], clinical information by the self-administered comorbidity questionnaire [99], and out-of-pocket expenditures by the Patient Self-Administered Financial Expenditure questionnaire [100]. These measurements will be taken 1 week after a surgery or after the second anti-cancer treatment (T0) at six and 12 months (T1 and T2), following International Consortium for Health Outcomes Measurement (ICHOM) guidelines [25, 101–103]. Although the ICHOM recommendations suggest a baseline at diagnosis at the physician’s office, our choice of T0 is intended to capture outcomes associated with “exposure” to relational coordination (RC) and to avoid adding to the potential distress of some PLCs by asking them to participate in a study right when they are receiving their diagnosis. Sociodemographic, clinical, and organizational data characterizing the sample that are potentially confounding variables will be collected according to ICHOM recommendations: sex, age, income, education, marital status, postal code, and self-reported comorbidities. As for the size of the PLC sample, a total of 356 respondents will have to be recruited to detect an average difference of 0.08 with a standard deviation of 0.4 and a correlation of 0.1 for health-related quality of life between measurement times [104]. A response rate of 70% is being assumed, based on our previous studies [12, 47].

#### Clinical records

Clinical results will be obtained from patient medical records, including the Outil de dépistage de la détresse (screening for distress tool – ODD) used in current practice in Quebec [105]. The ODD includes three scales: 1) the Distress Thermometer (DT) [106], a visual distress scale rated from 0 to 10 (10 being the highest level); 2) the revised Edmonton Symptom Assessment Scale (ESAS-R) to assess nine common cancer symptoms (pain, fatigue, drowsiness, nausea, anorexia, dyspnea, depression, anxiety, well-being) on a scale of 0 to 10 (10 being the worst manifestation of the symptom) [107, 108]; and 3) the Canadian Problem Checklist consisting of 24 items in the version promoted in Quebec [105]. The ODD data, including whether or not there is an active intervention, will be collected through a review of the patient record. There are different ways to clinically interpret these items, but we have selected the following scale: mild < 3, moderate = 4–6 and severe ≥7 [105].

#### Administrative data

The costs associated with the use of services will be calculated over a one-year period as with QALY measurements. They are in two areas: costs to the healthcare system and those incurred by PLCs [109]. Costs to the healthcare system include: hospitalizations, treatments and outpatient oncology and emergency room visits, drugs reimbursed by the Régie d’assurance-maladie du Québec (RAMQ) (Quebec’s health insurance plan agency), and physician fees. These costs will be calculated based on data from RAMQ’s medical and pharmaceutical services databases and the Quebec ministry of health and social services (MSSS) (MED-ECHO database). Unit costs for each type of service will be calculated from the MSSS’s annual budget and activity reports (AS-471, AS-478) submitted by each healthcare facility in Quebec. The average cost for care to PLCs (medical and general costs) can therefore be determined from the financial statements of each case in the study based on public reports. Physician fees, which include all medical procedures invoiced by physicians to the RAMQ that are not included in these reports, will be captured from the RAMQ along with the relative intensity levels of resources used. These data will provide an individual cost estimate for hospitalizations. Unit costs of medical services will be calculated for the most recent full fiscal year using a methodology published by two of the co-investigators [110]. Costs for outpatient oncology clinic visits and emergency room visits will be assessed on a cost-per-visit basis. Costs for hospitalizations include costs for hospital stay (cost per day) and day surgeries (cost per surgery). The RAMQ’s pharmaceutical services database will be used as a source for the costs of covered drugs.

### Quantitative analyses

The validity of quantitative results is based on the use of questionnaires with demonstrated psychometric qualities and implementation of the analysis plan under the guidance of co-investigators with solid expertise. Statistical analyses will be performed using IBM SPSS Statistics version 25 [111] and SAS 9.4 [112]. Values of *p* < 0.05 will be considered significant. Missing data for the variables will be imputed using the multiple imputation technique. Descriptive statistics will be used to summarize socio-demographic and clinical variables for PLCs and professional experience variables for care providers at each site. Student tests or non-parametric tests such as the Mann-Whitney test and ANOVA tests will be used for continuous variables. For categorical variables, square Chi-square tests will be used to identify differences between groups. Univariate and multivariable linear and logistic regression analyses will be conducted to explore the relationship between CG (mini-PEC and RC) and PLC characteristics, provider characteristics, and other site characteristics. We will use the strategy of Labbé et al. to identify the breaking points of clinically significant characteristics [104]. For the care experience, we will use the breakpoints between a positive vs. less positive experience used in our previous work [12]. Subsequently, multiple regression analyses will be conducted to determine the association between the independent variable (RC + mini-PEC) and the dependent variables (four reactivity subscales, three ODD dimensions, EQ-5D-5 L-EVA) by controlling for potentially confounding variables (PLC and site characteristics). The Incremental Cost-Effectiveness Ratio (ICER = ∆Cost /∆Effectiveness) will be estimated using a multivariate bootstrap, and cost-effectiveness acceptability curves will be constructed.

### Integrated knowledge translation

Our approach to intervention research in partnership with field actors, combined with the principles of realist evaluation, is based on a process of two-way exchange of knowledge and expertise between producers and users of research results involved in defining problems and constructing solutions [81, 113, 114]. This approach is strategically chosen because of its potential to accelerate the visibility of gains [81, 113], the positive influence it is recognized to have on the adoption of new practices based on research evidence, as well as its potential contribution to the management of complex problems [115, 116].

In order to translate the intermediate and final results of the study into impacts in the community, a knowledge transfer plan has been developed. The specific activities of our study are shown in Table 2. The components of the Knowledge to Action framework are the cornerstone for updating our IKT plan [81]. IKT is a dynamic and recursive process that encompasses the synthesis, dissemination, exchange and application of knowledge to improve the delivery of care and strengthen the healthcare system [117]. This process will be part of a complex network of producer–user interactions, whose intensity, complexity, and degree of engagement can be adapted according to the results we obtain and the particular needs of each user.

**Table 2:** Integrated knowledge translation activities

Target	Knowledge translation activities integrated into research
On-site collaborators, including persons living with and beyond cancer, clinicians, healthcare managers, and policy makers	• Announcement of the funding of the study during the Quebec Cancer Program Symposium • Strategic meetings with knowledge users (e.g. opinion leaders, collaborators) using bidirectional exchanges, deliberative process and reflexive approach throughout the research process to ensure early detection of obstacles, facilitating elements, controversies, and solutions • Access to the research team for on-site collaborators • Diffusion of newsletters or brief reports every 4 months reporting study progress and learnings • Discussions around the intermediate findings of the study in site meetings
Academic communities	• Involvement of junior researchers in teaching cancer care delivery: graduate students, physicians and health professionals • Diffusion of study results in classes lectured by the study researchers and collaborators in six different universities • Contribution to training and continuing education of health professionals • Mobilization of research networks in Quebec and Canada
Large scale	• Mobilization of partner networks: *Direction générale de cancérologie* (National Cancer Directorate), cancer networks, *Institut national d’excellence en santé et en services sociaux* (National Institute for Excellence in Health and Social Services) • Access to university and interuniversity networks: Quebec Network on Nursing Intervention Research (RRISIQ), Chaire de recherche sur la qualité et la sécurité des soins aux personnes atteintes de cancer de l’Université de Sherbrooke, Chaire Santé et Territoire du Groupe de recherche Asclépios de l’Université Clermont-Auvergne • Presentations in conferences: Multinational Association of Supportive Care in Cancer (MASCC), Canada’s Applied Research in Cancer Control Conference, Union for International Cancer Control (UICC), ASCO Palliative and Supportive Care in Oncology Symposium • Publication of the results on the website of Prof. Tremblay’s Research Chair to allow continuing knowledge transfer about the issues pertaining to collaborative governance • Publications in open access journals
Dissemination	• Creation and use of communication tools to disseminate the results of the study, adapted to target audiences, including summary documents (research notes or policy briefs) for policy makers, clinicians, and the general public.

Our interdisciplinary team (medicine, nursing, organization/management of services, sociology, management sciences, epidemiology, economics) is composed of leaders with the ability to reach governing actors of the QCN in Quebec. Knowledge mobilization has already begun with the composition of our team during the discussions to continue to work together on developing the QCN and making it sustainable. Knowledge mobilization is approached as a “contact sport” whose strategies are organized “around the water cooler” [118]. This metaphor illustrates the importance of creating relational spaces (virtual or face-to-face) between producers and users of research through a deliberative exchange process [119]. A reflexive approach and the presence of opinion leaders during these exchanges make it possible to clearly identify the representations that stakeholders have of the mechanisms of CG and its outcomes, and to identify realistic actions.

## Discussion

The sometimes contradictory findings on networks’ outcomes can be explained in part by the conceptual and methodological challenges of establishing causal links between integrated network operation and its outcomes on the quality of services or even population health [40]. Empirical studies, including ours, show variations in network implementation and degree of integration with mixed and sometimes unanticipated outcomes without having captured the sequence of mechanisms that explain these results [10, 40, 72]. One of the reasons is the lack of data on governance in these networks. In addition, most network studies are conducted over the short term, although it is recognized that interventions at the interface between clinic and organization take time [10].

Focusing on a CG model and its outcomes on the provision of care, our study responds to the challenge of innovating to address the issues of quality of care for PLCs, namely the increasing number of people receiving a new cancer diagnosis, the increasing number of those who survive it, and maintaining access to quality care in a context of scarce human resources and rising treatment costs [1]. It takes into account the difficulty of achieving the outcomes envisioned in cancer policies and programs [120].

Because of its pragmatic approach with close interaction between producers and users of research results, our study offers a strong potential for impact by making the QCN a learning organization [3]. The potential impact of our study on the network’s capacity for transformation will be observed at all three levels of the QCN’s care delivery: political, organizational, and clinical.

At the political level, the QCN’s capacity to transform the supply of care refers to the way in which government actors organize themselves to ensure that the national cancer control plan is implemented in a way that solves the problems of the supply of care and services to PLCs. However, this capacity depends on a wide variety of expertise from biology to sociology and from economics to epidemiology [121], expertise contributed by the members of our team that will have an impact on the ability to ensure the development of the QCN.

At the organizational level, managers have an important role to play in translating the elements of the national cancer plan into the specificities of their environment. Our study of CG will have an impact on four important aspects of their work in institutionalizing the network practices [122] promoted in the national cancer plan: 1) structural work: efforts to formalize roles, operating modes, resource allocation; 2) conceptual work: values, norms, and systems of representation; 3) operational work: concrete actions to guide clinicians’ behaviours and clinical practices; and 4) relational work: development of relationships, trust and collaboration among professionals involved in the provision of PLC care (encompassing the other three aspects) [48]. Our study on CG will provide access to data that can guide how managers carry out these aspects of their work.

At the clinical level, our study is the first to provide data on CG mechanisms where clinicians and PLCs are involved with government stakeholders to implement the national cancer plan. The study will impact the development of these new roles with the potential to reduce the gap between what is prescribed at the policy level (macro) and the delivery of care and services (micro) for which there is very little cancer-specific research data. Ultimately, our study will have an impact on organizations’ practices regarding the ratio of results to costs.

### Study challenges and mitigation strategies

There are some operational challenges in conducting the study. Different means have been devised to overcome these challenges.

The efforts required to maintain cohesion of the interdisciplinary research team, which includes several researchers and collaborators in different universities and research centres, should not be underestimated in this study involving a diversity of expertise. A steering committee (coordination and monitoring) and an advisory committee (interpretation of intermediate results) will be set up and a systematic evaluation of the conduct of meetings [123] and the satisfaction of participating in a community of practice [124] will make it possible to act to maintain interest. In addition, the study will be coordinated by a research professional.

The engagement and mobilization of health care providers in research, which is critical to achieving the objectives, is a challenge in a healthcare system undergoing transformation. Measures will be implemented to retain participation of care providers in the study, including information sessions on the benefits of the study, management support to facilitate participation, links established during the initial study, and monetary compensation for participation.

As for PLCs, the main challenge to their participation is likely to be the time and effort (availability, duration) required to participate in the data collection stages. They will be able to choose the time and place for the individual interviews. Particular care has been taken to select short and easy-to-use questionnaires for PLCs. In addition, the importance that sharing their experience represents as a contribution to the study will be highlighted, and monetary compensation will be offered in recognition of this contribution.

The large amount and diversity of data [125] could also pose challenges in terms of information storage and management. The QDA Miner, SPSS, and SAS software will facilitate the management, analysis and integration of qualitative, quantitative, and mixed data.

## Conclusion

With significant effort and resources being devoted to cancer networks, it is imperative to produce robust and rapidly usable research results to support decision-making on its future. The study of CG in cancer networks will contribute to an integrated understanding of vertical coordination between the levels of governance: between what is prescribed at the political level and what is actually happening in care and service teams, as well as horizontal coordination (between and within teams). This is a major challenge for the constant evolution of cancer networks and to ensure the sustainability of their benefits. Finally, it is important to determine whether the dynamics of collective governance of the network provide added value, i.e. better care outcomes for every dollar invested.

## Supplementary information

**Additional file 1: **Cancer network collaborative governance framework. **Figure S1.** Cancer network collaborative governance framework. **Table S1.** Concepts and definitions pertaining to Emerson’s collaborative governance framework and their contextualization to cancer networks. **Table S2.** Dimensions, components, and definitions of Emerson’s collaborative governance framework and their contextualization to cancer networks.

**Additional file 2: **Operationalization of variables. **Table S1.** List and description of quantitative data collection questionnaires.

## Data Availability

The datasets generated and/or analyzed during the current study will be available from the corresponding author on reasonable request. All data generated or analyzed during this study will be included in upcoming published articles.

## Abbreviations

C: Context
CG: Collaborative governance
M: Mechanism
O: Outcome
PLC: Person living with and beyond cancer
QALY: Quality-adjusted life years
QCN: Quebec Cancer Network
QUAL: Qualitative
QUAN: Quantitative
RC: Relational coordination
