# Supplementary material for: Collaborative governance in the Quebec Cancer Network: a realist evaluation of emerging mechanisms of institutionalization, multi-level governance, and value creation using a longitudinal multiple case study design
Source: BMC Health Serv Res. 2019 Oct 25;19:752. doi: 10.1186/s12913-019-4586-z (PMC6814997; doi:10.1186/s12913-019-4586-z)
Supplement: Supplementary file 1 — Additional file 1: Cancer network collaborative governance framework. Figure S1. Cancer network collaborative governance framework. Table S1. Concepts and definitions pertaining to Emerson’s collaborative governance framework and their contextualization to cancer networks. Table S2. Dimensions, components, and definitions of Emerson’s collaborative governance framework and their contextualization to cancer networks. [file 12913_2019_4586_MOESM1_ESM.docx]

**Additional material 1: Cancer network collective governance framework**

The study builds upon a cancer network collaborative governance framework adapted from the seminal work of Ansell and Gash [[1](#_ENREF_1)] and Emerson et al. [[2](#_ENREF_2), [3](#_ENREF_3)]. The definitions of the model components are useful for sharing a common representation of collaborative governance between knowledge producers and users, for guiding the operationalization of study variables, and for selecting appropriate data collection tools.

Our analytical framework is based on a broad synthesis of knowledge and is intended to be an integrative framework. It proposes: (1) to place the analysis of governance mechanisms in their context (availability of resources, legal context, socioeconomic context, power relations, past cancer control outcomes); (2) to analyze governance mechanisms as a set of implicit and explicit principles (rules, decision-making procedures), around which stakeholders in cancer control converge.

More specifically, the factors in the initial context that determine the effectiveness of governance mechanisms will be examined (perception of interdependencies, leadership, incentives), as well as the mechanisms of coordination dynamics. Coordination dynamics consist of three interrelated components: stakeholder engagement (clinicians, managers, policy makers, people affected by cancer), shared motivations and capacities for joint action between cancer teams and primary care providers. The analysis of stakeholder engagement invites us to look at mechanisms for building shared interests and a common vision, resolving controversies, and building consensus on actions to be taken. The understanding of shared motivations focuses on mechanisms for building trust and legitimizing teamwork. Finally, capacity for joint action refers to all the elements mobilized to generate potentially effective action, such as the procedural arrangements (protocols, structures, regulations) necessary to manage interactions, leadership, knowledge and resources. Depending on the context, coordination dynamics have the potential to produce practices or actions (outputs) that concretize the objectives of collaborative governance. For example, practices such as monitoring the deployment of the Quebec Cancer Network, redistributing professional and financial resources, developing new management practices and controlling costs can produce concrete results (outcomes) in terms of improving the response to the needs and preferences of people affected by cancer. These results can, in turn, feed into a transformation of the context and the dynamics of coordination through adaptation following the lessons learned. Overall, cross-boundary collaboration is expected to occur at multiple levels of decision-making in the cancer network.

**Figure 1 -** Cancer network collaborative governance framework

*Legend:*

The framework has been adapted from Emerson [[3](#_ENREF_3), [2](#_ENREF_2)] and integrated with Pawson and Tilley’s concepts [[4](#_ENREF_4)].

**Table 1 -** Concepts and definitions pertaining to Emerson’s collaborative governance framework and their contextualization to cancer networks

| **Concepts** | **Emerson’s original definitions [**[**3**](#_ENREF_3)**]** | **Contextualization to cancer networks (Tremblay)** |
| --- | --- | --- |
| **Cross-boundary collaboration** | Activity of collaboration between people from different organizations, sectors, or jurisdictions. (p. 230) | Activity of collaboration between people from different organizations, including public health agencies, ministries of health and social services, public health establishments, public and private clinics including primary care, community service and resource organizations. |
| **Collaborative governance** | Processes and structures of public policy decision making and management that engage people across the boundaries of public agencies, levels of government, and/or the public, private for-profit, and civic spheres to carry out a public purpose that could not otherwise be accomplished. (p. 229) | Processes and structures of the national cancer network partnering with persons living with and beyond cancer that engage people across the organizational boundaries and the national, regional and local levels of the network (ministry of health, public health establishments, public and private clinics including primary care providers, community service and resource organizations) to carry out the national cancer plan mission and goals. |
| **Collaborative governance regimes** | Modes of, or systems for, public decision making in which cross-boundary collaboration represents the prevailing pattern of behavior and activity between autonomous participants who have come together to achieve some collective purpose defined by one or more target goals. (p. 230)  Coordination dynamics, along with collaborative actions, constitute collaborative governance regimes. | Committees for decision making on specific cancer network issues in which collaboration across organizational boundaries and the national, regional and local levels represents the prevailing pattern of behavior and activity between autonomous health managers, professionals, and organizations collaborating to achieve cancer networks’ mission and goals.  Coordination dynamics and collaborative actions drive cross-boundary and multilevel collaborative committees implemented within and across cancer networks. |
| **Externally directed collaborative governance regimes** | Formed by initiating leaders with a more **removed or indirect stake** in addressing extensive, recurring policy challenges, and who are situated in agencies or organizations with explicit, concentrated authority in the subject policy area.  These collaborative governance regimes develop through a **formally structured** approach that creates incentives or mandates for participants  Participants’ collective autonomy is **constrained or directed** to some extent by the authorized collaborative structure. (p. 230) | Formed by leaders of national and local cancer control programs partnering with persons living with and beyond cancer to address the challenges of the fight against cancer, situated in national health policy bodies (e.g. ministry of health) that have explicit, concentrated authority in coordinating the fight against cancer.  Those programs are externally directed collaborative governance regimes that develop through formally structured cancer networks supported by mandates and incentives for participants.  Participants’ collective autonomy is constrained or directed to some extent by the mandated national and local coordination committees (including the medical and clinical-administrative co-managers, the cancer program director, and a representative of persons living with and beyond cancer), authorized communities of practice and formalized consultative committees. |

**Table 2 -** Dimensions, components and definitions of Emerson’s collaborative governance framework and their contextualization to cancer networks

| **Dimensions** | **Components** | **Emerson’s original definitions** [[3](#_ENREF_3)] | **Contextualization to cancer networks (Tremblay)** |
| --- | --- | --- | --- |
| **Context** |  | Broad and dynamic set of surrounding conditions that create opportunities and constraints for initiating and sustaining collaborative governance regimes. (p. 232) Context includes: | Broad and dynamic set of surrounding conditions that create opportunities and constraints for initiating and sustaining national and local cancer control programs. |
|  |  | - Legal frameworks | - Health laws and acts, drug regulations, acts and regulations framing the practice of health professions |
|  |  | - Policy frameworks | - National cancer plan |
|  |  | - Public resources and service conditions | - Publicly funded healthcare system |
|  |  | - Socioeconomic characteristics | - Developed country |
|  |  | - Cultural characteristics | - Heterogeneity of ethnic, disciplinary, institutional, generational cultures, etc. |
|  |  | - Network characteristics | - Cancer network hybridity (mandated and coordinated) |
|  |  | - Political dynamics | - National cancer program top-down prescription and 2015 major healthcare reform |
|  |  | - Power relations | - Hierarchical relations, medical power, professional bureaucracy, scopes of practice, interprofessional practice |
|  |  | - History of conflict | - Cancer crisis, competition between health establishments |
| **Drivers**  **(adapted to “Initial context”)** |  | Factors that help propel the creation of a collaborative governance regime. (p. 230) | Initial contextual factors that help propel the creation of dedicated spaces (formal committees) for cross-boundary and multilevel collaborations within and across cancer networks to provide integrated care and services to persons living with and beyond cancer:   - shared values posited through the National cancer program; - institutionalization of cancer patient contribution in the governance structure. |
|  | - Initiating leadership | Presence and actions of a person or core group that stimulates interest in and instigates preliminary discussions about creating a collaborative endeavor. (p. 230) | A shared leadership approach involving healthcare managers and professionals who stimulate interest in the national cancer plan and instigate its translation into practices. |
| **Dimensions** | **Components** | **Emerson’s original definitions** [[3](#_ENREF_3)] | **Contextualization to cancer networks (Tremblay)** |
| **Drivers**  **(adapted to “Initial context”)**  ***(continued)*** | - Conse-quential incentives | Internal issues, resource needs, interests, or opportunities and external situational or institutional crises, threats, or opportunities that must be addressed to mitigate salient risk or advance desired conditions for key stakeholders and the broader public. (p. 230) | Internal issues, resource needs, interests, or opportunities and external situational or institutional crises, threats, or opportunities that must be addressed to   - mitigate salient risk for health professionals and managers promoting team resiliency; or - advance desired conditions for persons living with and beyond cancer. |
|  | - Inter-dependence | Acknowledged necessity of mutual reliance between groups and organizations to accomplish desired goals. (p. 231) | Acknowledged necessity of mutual interdependences between governance committees, care teams, and health care organizations to provide integrated care and services to persons living with and beyond cancer. |
|  | - Uncertainty | Situations of doubt and limited information about future conditions, events, availability of resources, or decisions by other actors. (p. 232) | Situations of doubt and limited information about the “cancer crisis” (e.g. the shrinking cancer care workforce, the growing complexity of cancer care delivery, changing guidelines). |
| **Collabora-tion**  **Dynamics (adapted to “Coordination Dynamics”)** |  | Progressive and iterative cycling of three components which takes place over time between collaborative governance regime participants and between participants and their parent organizations. (p. 229) | Progressive and iterative cycling of three components which takes place over time between the participants of cross-boundary multilevel cancer committees and between participants and their parent cancer networks. |
|  | - Principled engagement | Behavioral interactions between collaborative governance regime participants. (p. 231) | Behavioral interactions between participants of cross-boundary multilevel cancer committees. |
|  | - Shared motivations | Interpersonal relations between collaborative governance regime participants. (p. 231) | Interpersonal relations between participants in cross-boundary multilevel cancer committees. |
|  | - Capacity for joint action | Functional assets available to the collaborative governance regime. (p. 229) | Functional assets available to the cross-boundary multilevel cancer committees. |
|  |  |  |  |
|  |  |  |  |
| **Dimensions** | **Components** | **Emerson’s original definitions** [[3](#_ENREF_3)] | **Contextualization to cancer networks (Tremblay)** |
| **Collabora-tive actions** |  | Intentional efforts taken as a consequence of the shared theory of change (or action) developed by the collaborative governance regime during collaboration dynamics to achieve its collective purpose and target goals. (p. 229) | Intentional efforts taken as a consequence of shared action plans and rationales developed by the cross-boundary multilevel cancer committees during collaboration dynamics to achieve its mission, mandate, and target goals. |
| **Outputs [**[**2**](#_ENREF_2)**]** |  | Intermediate actions produced through collaboration conducive to outcomes |  |
|  |  | Securing endorsements |  |
|  |  | Enacting policy, Law Rule | Enacting framework for partnering with persons living with and beyond cancer (activated) |
|  |  | Marshalling resources | Redistributing resources (activated) |
|  |  | Deploying staff |  |
|  |  | Siting permitting |  |
|  |  | Building / Cleaning up |  |
|  |  | Enacting new management practice | Enacting new governance practices |
|  |  | Monitoring implementation |  |
|  |  | Enforcing compliance |  |
| **Mechanismsof cross-boundary collabora-tion** |  | Resources and reasonings mobilized by participants concerned by a specific program in a specific context to bring about a set of intended outcomes. [[5](#_ENREF_5)] | Resources mobilized and reasoning deployed by the governance committees, care teams, and health care organizations collaborating in the cancer networks to bring about a set intended outcomes, including but not restricted to those posited in the national and the local cancer control programs. Mechanisms could include collaboration dynamics, collaborative actions and outputs. |
|  |  |  |  |
|  |  |  |  |
| **Dimensions** | **Components** | **Emerson’s original definitions** [[3](#_ENREF_3)] | **Contextualization to cancer networks (Tremblay)** |
| **Outcomes** |  | Intermediate changes in conditions necessary to reach target goals and the end effects of accomplishing these goals. Outcomes are a consequence of collaborative actions. (p. 231) | Idem |
|  | - Process performance | Level of functioning of collaboration dynamics that emerges from the combined interactions of the components of coordination dynamics (principled engagement, shared motivation, and the capacity for joint action). (p. 231) | Idem |
|  | - Productivity performance | Outcomes and adaptation generated by actions (or outputs) of the collaborative governance regime (p. 231) | Network-based practices of the cross-boundary multilevel cancer committees and the outcomes and adaptation they generate. |
|  | - Monitoring | Not applicable | Documenting indicators to assess whether performance targets are met within participant organizations. Monitoring is performed by actors of the cancer networks and the results are reported to members of the cross-boundary multilevel cancer committees. |
|  | - Learning | Not applicable | Uptake and use of performance results to make decisions pertaining to adaptations needed to reach the objectives of national and local action plans against cancer. Learning can occur within the cross-boundary multilevel cancer committees and within participant organizations. |
|  | - Adaptation | Transformative changes, or small but significant adjustments, that are made in response to the outcomes of collaborative actions. Adaptation can occur within the collaborative governance regime, between the participant organizations, and in the target goals. (p. 229) | Transformative changes, or small but significant adjustments, that are made in response to the outcomes of collaborative actions. Adaptation can occur within the cross-boundary multilevel cancer committees, between the participant organizations, and in the target goals. |

**References:**

1. Ansell C, Gash A. Collaborative governance in theory and practice. J Public Adm Res Theory. 2007; 18 (4): 543-71.

2. Emerson K, Nabatchi T, Balogh S. An integrative framework for collaborative governance. J Public Adm Res Theory. 2011; 22 (1): 1-29.

3. Emerson K, Nabatchi T. Collaborative governance regime. Washington, DC: Georgetown University Press; 2015.

4. Pawson R, Tilley N. Realist evaluation. Magenta Text. 2004. Accessed May 16 2019.

5. Dalkin S, Greenhalgh J, Jones D, Cunningham B, Lhussier M. What's in a mechanism? Development of a key concept in realist evaluation. Implementation science : IS. 2015; 10 (1): 49.
