# Supplementary material for: Collaborative governance in the Quebec Cancer Network: a realist evaluation of emerging mechanisms of institutionalization, multi-level governance, and value creation using a longitudinal multiple case study design
Source: BMC Health Serv Res. 2019 Oct 25;19:752. doi: 10.1186/s12913-019-4586-z (PMC6814997; doi:10.1186/s12913-019-4586-z)
Supplement: Supplementary file 2 — Additional file 2: Operationalization of variables. Table S1. List and description of quantitative data collection questionnaires. [file 12913_2019_4586_MOESM2_ESM.docx]

**Additional file 2: Operationalization of variables**

The outcomes of local cancer networks will be evaluated based on the experiences of caregivers and persons living with and beyond cancer (PLC) and the monthly out-of-pocket expenditures of PLCs. Data will be collected through self-administered questionnaires completed by caregivers and PLCs. Table 1 lists and describes the questionnaires that will be used.

**Health care provider questionnaires: organizational outcomes**

Relational coordination is defined as “a mutually reinforcing process of communicating and relating for the purpose of task integration and is useful for coordinating work that is highly interdependent, uncertain, and time constrained.” The relational coordination measure is operationalized by the Relational Coordination questionnaire [[1](#_ENREF_1)]. It is one of few “unbounded” instruments, suitable for measuring relational coordination within and between teams as well as between levels of a healthcare system. Its use is appropriate when the work is characterized by high levels of interdependence, uncertainty and time pressure, which are intrinsic to oncology. The outcomes that are associated with relational coordination are: quality of care, experience of care, efficiency (including cost control), and professional satisfaction [[1](#_ENREF_1)].

Two additional questionnaires were chosen for their psychometric qualities and short completion time (3-5 minutes each). The dynamics of collaboration within and between care teams are operationalized by the short version of the Practice Environment Checklist mini-PEC (5 items, α=0.82) [[2-4](#_ENREF_2)]. The first subscale of Rousseau’s teamwork questionnaire measures interpersonal support between team members [[5](#_ENREF_5)]. The remainder of the questionnaire covers demographic and work experience data.

**Persons living with and beyond cancer:**

***Patient-reported experience* *questionnaires***

Three questionnaires were selected to measure patient reported experience and outcomes.

The care experience of people living with and beyond cancer (PLC) is operationalized by the Health System Responsiveness questionnaire [[6](#_ENREF_6)], one of the key indicators of health system performance [[7](#_ENREF_7)], translated into French and adapted for cancer care by Tremblay [[8](#_ENREF_8)].

It includes 19 items grouped into four subscales measured from 1 to 4 (1=never; 4 always): promptness of access (4 items, α=0.77), person-centred response (5 items, α=67), quality of communication (5 items, α=0.85), quality of the care environment (5 items, α=0.64). In order to be useful to decision-makers, a positive experience represents excellence, or the maximum level (4=always) at each question on a subscale. The PLCs’ perception of their patient-reported experience measure (PREM) will be taken in T2 only and will cover the previous 12 months [[9](#_ENREF_9)]. Evaluation of the care experience is imperative to improve provision of care centred on PLCs [[10](#_ENREF_10)].

Quality of life is measured using the EuroQoL EQ-5D-5L (α=0.85) [[11](#_ENREF_11)] which has good construct validity and discriminatory power [[12](#_ENREF_12)].

This instrument has 5 dimensions (mobility, autonomy, current activities, pain/discomfort, anxiety/depression) on a 5-level scale (1=no problem; 5=extreme problem). It is complemented by a visual analogue scale (EQ-5D-ÉVA) on perceived health status from 0 to 100 (0 being the worst health status imaginable). By combining the levels for each dimension, it is possible to identify 3,125 health profiles. From these results, a value index is created based on a weighting according to the preferences of the Canadian population [[13](#_ENREF_13), [14](#_ENREF_14)]. EQ-5D-5L is often used in cost analyses, particularly in the area of cancer in Canada [[15](#_ENREF_15)]. Looking at the difference in the results of the EQ-5D-5L at different measurement times, it is possible to determine the quantity and quality of life (Quality-Adjusted Life Years (QALY)) [[16](#_ENREF_16)] by calculating the area under the curve between different measurements. QALYs are used to evaluate the effectiveness of interventions (CG in our study) in terms of cost. This evidence is crucial for allocating resources and making decisions about how to organize patient-centred services [[17](#_ENREF_17)] while controlling costs.

The Self-Administered Comorbidity Questionnaire (SCQ) will be used to document other health problems among PLCs. This questionnaire includes 12 health conditions and 3 conditions (Do you have this problem; are you receiving treatment for this problem; does this problem limit your activities) on a Yes=1/No=0 scale (score between 0-36) [[18](#_ENREF_18)], support from a loved one, type of cancer, delay since diagnosis, type of treatment, response to treatment, occurrence of early complications in T1.

Sociodemographic data and other clinical information characterizing the sample that are potentially confounding variables will be collected according to ICHOM recommendations: gender, age, education level, socioeconomic status, postal code.

***Out-of-pocket expenditures questionnaire***

The self-reported monthly out-of-pocket expenditures of the participating PLCs will be calculated in the 4 cases under study. Non-covered health services (alternative therapies, psychotherapy, physiotherapy, drugs obtained from a community pharmacy with or without a prescription, special diet, equipment, health insurance fees) and non-medical costs (travel between home and place of delivery at Canada Revenue Agency rates, absence from work of the patient and/or family member for treatment and follow-up visits) will be taken into account [[19](#_ENREF_19)]. The Patient Self-Administered Financial Expenditure (P-SAFE) questionnaire, adapted for Canada, will be used to collect data while PLCs are receiving their treatments and to measure PLCs’ perception of the financial burden of cancer [[20-22](#_ENREF_20)]. The data will be validated from the patient file review.

**Table 1 - List and description of quantitative data collection questionnaires**

| **Outcomes** | **Instruments** | **Dimensions** | **Number of items** | **Response scale** | **Cronbach alpha** |
| --- | --- | --- | --- | --- | --- |
| **Professional-reported experience** | - Relational coordination questionnaire [[23](#_ENREF_23), [24](#_ENREF_24), [1](#_ENREF_1)] | Relationships:   - Shared goals - Shared knowledge - Mutual respect   Communication:   - Frequent - Timely - Accurate - Problem-solving | - 1 item per dimension | - 5 point (1=never; 5=always) | - 0.80 |
|  | - Mini Practice Environment Checklist (Mini-PEC) [[4](#_ENREF_4)] | - Clinical team functioning | - 5 items | - 4 point (1=strongly disagree; 4=strongly agree) | - 0.89 |
|  | - Team functioning [[5](#_ENREF_5)] | - Interpersonal support | - 12 items | - 5 point (1=not true at all; 5=entirely true) | - 0.93 |
|  | - Sociodemographic and work information | - Gender; Age; Education and training; Profession; Work experience | - 12 items | - Varied scales - Fill-in fields | - Not applicable |
| **Patient-reported experience** | - Health System Responsiveness questionnaire [[9](#_ENREF_9), [8](#_ENREF_8), [6](#_ENREF_6)] | - Prompt access to care | - 4 items | - 4 point (1=never; 4=always) | - 0.77 |
|  |  | - Person-centred response | - 5 items | - 4 point (1=never; 4=always) | - 0.67 |
|  |  | - Patient-provider communication | - 5 items | - 4 point (1=never; 4=always) | - 0.85 |
|  |  | - Quality of the care environment (Quality of basic amenities) | - 5 items | - 4 point (1=never; 4=always) | - 0.64 |
|  |  | - Cancer service responsiveness | - 19 items | - 4 point (1=never; 4=always; and 1=not at all; 4=a lot) | - 0.90 |
|  | - EuroQoL EQ-5D-5L questionnaire and analogous visual scale   (EQ-5D-VAS) [[11](#_ENREF_11), [7](#_ENREF_7), [15](#_ENREF_15), [13](#_ENREF_13), [12](#_ENREF_12), [16](#_ENREF_16), [17](#_ENREF_17)] | - Mobility - Self-care - Usual activities - Pain / discomfort - Anxiety / Depression | - 1 item per dimension | - 5 point | - 0.85 |
|  |  | - Global health today | - Thermometer | - 0 - 100 | - Not applicable |
|  | - Comorbidity questionnaire (SCQ) [[18](#_ENREF_18)] | - 13 chronic conditions (including cancer) - 2 other conditions | - 3 items per condition | - Dichotomic (0=no; 1=yes) | - Not applicable - Intraclass correlation coefficient=0.94 |
|  | - Other clinical information | - Cancer diagnosis and treatment; Use of health care and services | - 25 items | - Varied scales - Fill-in fields | - Not applicable |
|  | - Sociodemographic information | - Gender; Education level; Socioeconomic status; Age; Postal code | - 5 items | - Varied scales - Fill-in fields | - Not applicable |
| **Out-of-pocket expenditures** | - Patient Self-Administered Financial Expenditure questionnaire (P-SAFE) [[22](#_ENREF_22), [21](#_ENREF_21), [20](#_ENREF_20)] | - Insurance plans and extra costs - Time away from work - Other costs - Individual clinical and socioeconomic characteristics | - 23 questions: 16 multiple choices question + 7 varied format questions | - Varied scales - Tables and other formats with fill-in fields | - Not available |

**References**

1. Gittell JH, Beswick J, Goldmann D, Wallack SS. Teamwork methods for accountable care: relational coordination and TeamSTEPPS®. Health Care Manage Rev. 2015;40(2):116-25.

2. Jaén C, Crabtree B, Miller W. Clarification regarding the practice environment checklist. Fam Med. 2012;44(2):135.

3. Jaén CR, Crabtree BF, Palmer RF, Ferrer RL, Nutting PA, Miller WL et al. Methods for evaluating practice change toward a patient-centered medical home. Ann Fam Med. 2010;8 Suppl 1:S9-20; S92.

4. Lurie SJ, Schultz SH, Lamanna G. Assessing teamwork: a reliable five-question survey. Fam Med. 2011;43(10):731-4.

5. Rousseau V, Aubé C, Savoie A. Le fonctionnement interne des équipes de travail: conception et mesure. Canadian Journal of Behavioural Science / Revue canadienne des sciences du comportement. 2006;38(2):120-35.

6. Valentine NB, de Silva A, Kawabata K, Darby C, Murray CJL, Evans DB. Health system responsiveness: concepts, domains and operationalization. In: Murray CJL, Evans DB, editors. Health systems performance assessment: debates, methods and empiricism. Geneva: WHO; 2003. p. 573-96.

7. World Health Organization (WHO). The world health report 2000. health systems : improving performance. WHO, Geneva. 2000. https://[www.who.int/whr/2000/en/](http://www.who.int/whr/2000/en/). Accessed May 16 2019.

8. Tremblay D. La traduction d’une innovation organisationnelle dans les pratiques professionnelles de réseau: l’infirmière pivot en oncologie. Université de Montréal (Canada), Montréal, QC. 2008. https://papyrus.bib.umontreal.ca/xmlui/bitstream/handle/1866/6667/Tremblay_Dominique_2008_these.pdf?sequence=1&isAllowed=y. Accessed May 16 2019.

9. Tremblay D, Roberge D, Berbiche D. Determinants of patient-reported experience of cancer services responsiveness. BMC Health Serv Res. 2015;15:425.

10. Canadian Partnership Against Cancer. Cancer partners take action to deliver person-centred cancer care. Canadian Partnership Against Cancer, Toronto, ON. 2018. https://[www.partnershipagainstcancer.ca/news-events/news/article/cancer-partners-action-deliver-person-centred-cancer-care/](http://www.partnershipagainstcancer.ca/news-events/news/article/cancer-partners-action-deliver-person-centred-cancer-care/). Accessed May 16 2019.

11. van Reenen M, Janssen B. EQ-5D-5L user guide. Basic information on how to use the EQ-5D-5L instrument. EuroQol Research Foundation, The Netherlands. 2015. https://euroqol.org/wp-content/uploads/2016/09/EQ-5D-5L_UserGuide_2015.pdf. Accessed May 16 2019.

12. Janssen MF, Pickard AS, Golicki D, Gudex C, Niewada M, Scalone L et al. Measurement properties of the EQ-5D-5L compared to the EQ-5D-3L across eight patient groups: a multi-country study. Qual Life Res. 2013;22(7):1717-27.

13. Xie F, Pullenayegum E, Gaebel K, Bansback N, Bryan S, Ohinmaa A et al. A time trade-off-derived value set of the EQ-5D-5L for Canada. Med Care. 2016;54(1):98-105.

14. Bansback N, Tsuchiya A, Brazier J, Anis A. Canadian valuation of EQ-5D health states: preliminary value set and considerations for future valuation studies. PLoS ONE. 2012;7(2).

15. Labbé C, Leung Y, Silva Lemes JG, Stewart E, Brown C, Cosio AP et al. Real-world EQ5D health utility scores for patients with metastatic lung cancer by molecular alteration and response to therapy. Clin Lung Cancer. 2017;18(4):388-95.e4.

16. Garau M, Shah KK, Mason AR, Wang Q, Towse A, Drummond MF. Using QALYs in cancer. Pharmacoeco. 2011;29(8):673-85.

17. Smith MD, Drummond M, Brixner D. Moving the QALY forward: rationale for change. Value Health. 2009;12(Supplement 1):S1-S4.

18. Sangha O, Stucki G, Liang MH, Fossel AH, Katz JN. The self-administered comorbidity questionnaire: a new method to assess comorbidity for clinical and health services research. Arthritis Rheum. 2003;49(2):156-63.

19. Zafar SY, Peppercorn JM, Schrag D, Taylor DH, Goetzinger AM, Zhong X et al. The financial toxicity of cancer treatment: a pilot study assessing out-of-pocket expenses and the insured cancer patient’s experience. Oncologist. 2013;18(4):381-90.

20. Longo CJ, Bereza BG. A comparative analysis of monthly out-of-pocket costs for patients with breast cancer as compared with other common cancers in Ontario, Canada. Curr Oncol. 2011;18(1):e1-8.

21. Longo CJ, Deber R, Fitch M, Williams AP, D’Souza D. An examination of cancer patients’ monthly ‘out-of-pocket’ costs in Ontario, Canada. Eur J Cancer Care (Engl). 2007;16(6):500-7.

22. Longo CJ, Fitch M, Deber RB, Williams AP. Financial and family burden associated with cancer treatment in Ontario, Canada. Support Care Cancer. 2006;14(11):1077-85.

23. Gittell JH. Coordinating mechanisms in care provider groups: relational coordination as a mediator and input uncertainty as a moderator of performance effects. Management Science. 2002;48(11):1408-26.

24. Gittell JH, Seidner R, Wimbush J. A relational model of how high-performance work systems work. Organization Science. 2010;21(2):490-506.
